# Case Report: Whole exome sequencing identifies a novel frameshift insertion c.1325dupT (p.F442fsX2) in the tyrosine kinase domain of *BTK* gene in a young Indian individual with X-linked agammaglobulinemia

**DOI:** 10.12688/f1000research.9472.2

**Published:** 2017-08-31

**Authors:** Amit Rawat, Shamsudheen Karuthedath Vellarikkal, Ankit Verma, Rijith Jayarajan, Anju Gupta, Surjit Singh, Anita Chopra, Rajive Kumar, Vinod Scaria, Sridhar Sivasubbu

**Affiliations:** 1 Advanced Pediatrics Centre, Postgraduate Institute of Medical Education and Research, Chandigarh, 160012, India; 2 Academy of Scientific and Innovative Research (AcSIR), CSIR Institute of Genomics and Integrative Biology, New Delhi, 110025, India; 3 Genomics and Molecular Medicine Unit, CSIR Institute of Genomics and Integrative Biology, New Delhi, 110025, India; 4 Lab oncology, All India Institute of Medical Sciences, New Delhi, 110029, India; 5 GN Ramachandran Knowledge Center for Genome Informatics, CSIR Institute of Genomics and Integrative Biology, New Delhi, 110025, India

**Keywords:** X-linked agammaglobulinemia, Bruton's tyrosine kinase, Whole exome sequencing, Flow cytometry, B-lymphocytes

## Abstract

X-linked agammaglobulinemia (XLA) is an extremely rare inherited primary immunodeficiency characterized by recurrent bacterial infections, decrease in number of mature B cells and low serum immunoglobulins. XLA is caused by mutations in the gene encoding Bruton's tyrosine kinase. We report a case of a young Indian boy suspected to have XLA. Immunophenotyping was performed for the affected child using CD20, CD19 and CD3 antibodies. Whole exome sequencing was performed using trio-based approach. The variants were further analyzed using capillary sequencing in the trio as well as maternal grandmother. Initial immunophenotyping in the affected child showed decreased count of CD19+ B cells. To strengthen the clinical findings and confirm the diagnosis of XLA, we performed whole exome sequencing. Our analysis identified a novel frameshift insertion (c.1325dupT) in the *BTK* gene, which was further validated by Sanger sequencing. Our approach shows the potential in using whole exome sequencing to pinpoint the molecular lesion, enabling timely diagnosis and genetic counseling, and potentially offering prenatal genetic testing for the family.

## Introduction

Primary immunodeficiencies are congenital defects in the immune defence mechanisms of the host against invading pathogens. X-linked agammaglobulinemia (XLA) is a primary immunodeficiency disorder (OMIM# 300755) characterized by recurrent infections causing pneumonia, conjunctivitis, gastrointestinal infections, otitis media and sinopulmonary infections ^1^, which may require frequent hospitalization. The disease is extremely rare with an estimated prevalence of 3–6 per 1,000,000 males ^2^ and is inherited in an X-linked recessive manner. The disease arises from genetic defects, due to which the mature B lymphocytes are either low in number or completely absent in the bloodstream while also exhibiting a complete absence of serum immunoglobulins ^1^. The absence of immunoglobulins results in a compromised humoral immune response, which makes the affected individual extremely vulnerable to infections with encapsulated bacteria and enteroviruses. Molecular genetic studies have conclusively mapped the genetic locus of XLA to the gene encoding for the Bruton’s tyrosine kinase (BTK) ^3^. A comprehensive mutation database (BTKbase) lists over 700 unique mutations associated with XLA that affect the activity of BTK protein ^4^.

## Case report

**Figure 1.**
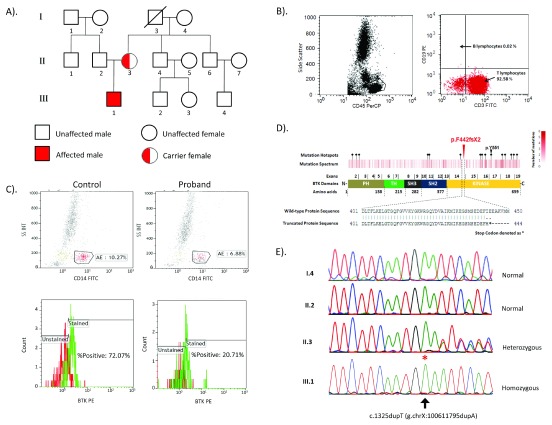
**A**). Pedigree of the family **B**). Flow cytometric immunophenotyping of peripheral blood lymphocytes gated on side scatter/CD45 plot. In CD19/CD3 plot, arrow depicts the count of B lymphocytes (CD19+) and T lymphocytes (CD3+). **C**). Flow cytometric estimation of BTK protein expression on CD14+ monocytes in control and proband. **D**). Heat map showing mutation spectrum corresponding to BTK protein structure and hotspots are marked with black diamond symbol. BTK domains (PH, TH, SH3, SH2 and Kinase) are marked in respective colors. The exons encoding specific region of domain and amino acids span for each domain is represented in schematic of BTK. A red triangle represents the novel variation p.F442fsX2, which lies in exon 14 and BTK kinase domain. The widely studied p.Y551 is also marked with black arrow. Pairwise alignment between wild type and truncated protein sequence is performed by using EMBOSS online software. **E**). The chromatogram depicts capillary sequencing results of c.1325dupT (p.F442fsX2) (marked with asterisks).

A five year old boy of north Indian origin, born out of a non-consanguineous marriage ( Figure 1A) presented to the hospital with headache, fever and a history of recurrent infections requiring hospitalization. The antenatal and perinatal periods were uneventful. His clinical history revealed that the child had been hospitalized for septicaemia and underwent treatment with intravenous (IV) antibiotics for 2 weeks at one year of age. Later, at 2.5 years, the child developed fever with swelling in right knee joint that was diagnosed as septic arthritis. The child was again hospitalized at 5 years of age for pyogenic meningitis. The culture of cerebrospinal fluid was found to be positive for *Pseudomonas aeruginosa*. On close examination, the tonsils were found to be absent and there was no peripheral lymphadenopathy. The immunoglobulin profile revealed serum level of IgG 50 mg/dl (200–700 mg/dl), IgA 5 mg/dl (40–200 mg/dl) and IgM 9 mg/dl (50–200 mg/dl). The hemogram showed hemoglobin concentration of 9.5 g/dl (11.5–15.5 g/dl), total leukocyte count 14800/cumm (6000–13500/cumm), platelet count 931000/cumm (150000–400000/cumm) and erythrocyte sedimentation rate ESR 18mm/1st hr (0–14 mm/1st hr).

The blood flow cytometric analysis of the affected child was performed to evaluate the status and count of mature B cells. The patient (III.1) has only CD3+ lymphocytes as observed on CD19/CD3 dot plot and there was complete absence of CD19+ cells (0.02%) ( Figure 1B). The staining for BTK protein on CD14+ monocytes showed decreased (20.71%) expression of BTK in proband as compared to control (72.07%) ( Figure 1C). This observation was consistent with the diagnosis of XLA ^5^. The patient had no family history of immunodeficiency and no such characteristics were present in any other family members.

The blood samples were collected and processed for genomic DNA isolation by salting out method ^6^. We performed the whole exome sequencing using trio-based approach (patient, mother and father). In brief, the whole-exome library was prepared using Nextera rapid capture expanded exome kit (Illumina Inc., USA) according to manufacturer’s standard protocol. Sequencing was performed on Illumina Hiseq2500 platform (Illumina Inc., USA) with 130bp paired-end reads. Reads were trimmed using Trimmomatic v0.33 ^7^ and aligned to reference genome hg19 (GRCh37) by Stampy v1.0.23 ^8^ along with BWA v0.7.12-r1039 ^9^. PCR duplicates were marked using Picard tools v1.127. Variations were called using Platypus v0.7.9 ^10^ and annotated using ANNOVAR ^11^. Analysis revealed a novel frameshift insertion c.1325dupT in exon 14 of the *BTK* gene. The mutation was found to be homozygous in child and heterozygous in mother. The identified mutation c.1325dupT has not yet been reported in the BTKbase ^4^ and absent in ExAC, 1000genome as well as internal control databases from South Asia and Middle East ( http://clingen.igib.res.in/almena), which confirms the novelty of the variation. The mutation evaluation by SIFT Indel tool ( http://sift.bii.a-star.edu.sg/www/SIFT_indels2.html, 12) was predicted to be damaging and caused nonsense mediated decay (confidence score 0.858). Further *in silico* analysis suggested that the mutation causes Isoleucine at 443 residue in BTK to be replaced by Histidine and introduces a premature stop codon at 444 residue, which lies in the kinase domain of the BTK protein ( Figure 1D).

The variation was further validated by PCR amplification of region encompassing the variation using specific primer sets (Forward primer: 5’-CCCCAAATGCTACTGAGATGGT-3’ and Reverse primer: 3’-CCTATTTCTACCCCAGTAGGGA-5’) with the annealing temperature of 59°C using Brazilian *taq* polymerase (Invitrogen, USA) according to manufacturer instruction. PCR products were purified using Qiaquick PCR purification kit (QIAGEN, Germany). Capillary sequencing was performed using BigDye-terminator chemistry on 3130xl Genetic Analyzer (Applied Biosystems, USA). Analysis revealed that the mutation was homozygous in child (III.1), heterozygous in mother (II.3) and absent in father (II.2) and maternal grandmother (I.4) ( Figure 1E).

## Discussion

XLA is a primary immunodeficiency disorder characterized by recurrent infections causing pneumonia, conjunctivitis, gastrointestinal infections, otitis media and sinopulmonary infections ^1^. Whole exome sequencing has been increasingly used to identify mutations in rare genetic diseases mainly due to the speed, cost and amenability as compared to traditional capillary sequencing ^13^. Recent reports have suggested the application of whole exome sequencing for mutation detection in a variety of primary immunodeficiency cases ^14, 15^.

In the present report, we performed whole exome sequencing using a trio-based approach for a child from an Indian family who presented to the clinic with the suspected diagnosis of XLA. The lack of readily available specific gene sequencing assays coupled with absence of a next-generation sequences (NGS) based targeted gene panels for XLA provided the impetus for attempting exome sequencing.

Our exome sequencing analysis revealed a novel frameshift insertion c.1325dupT in exon 14 of the *BTK* gene. The mutation was found to be homozygous in patient and heterozygous in unaffected mother, which was further validated by capillary sequencing. This confirmed the X-linked inheritance and carrier status of the mother for the mutation. The mutation was found to be absent in unaffected father and maternal grandmother. The identified mutation c.1325dupT was found to be novel and damaging due to truncation of the BTK at 444 residue of kinase domain. The flow cytometric analysis for BTK stained monocytes shows decreased expression of BTK in proband as compared to control ( Figure 1C). The mutation excludes functionally well characterized active site residue Y551 of the protein. Additionally, nonsense mutation at the codon Y425X, E441X, Q459X and Q497X is known to cause loss of kinase activity of BTK, which has been previously demonstrated using *in vitro* kinase activity assay in Japanese individuals ^16^. Since c.1325dupT (p.F442fsX2) lies in the vicinity of the above mentioned well studied codon positions, the effect of the mutation is expected to be damaging to BTK. Currently the patient is on intravenous immunoglobulin replacement therapy (15 g every 3–4 weekly) and is responding well. We could not avail the RNA samples to perform transcript analysis or functional studies.

In summary, our flow cytometry data and exome sequencing analysis are well correlated for confirming the diagnosis of XLA. The outcome from the present study strongly supports the pathogenicity of identified novel mutation in *BTK* gene.

## Consent

Written informed consent was obtained the parents of the child.

## Data availability

The data referenced by this article are under copyright with the following copyright statement: Copyright: © 2017 Rawat A et al.

The raw sequencing data are available at NCBI Sequence Read Archive ( http://www.ncbi.nlm.nih.gov/sra) with accession number SRR3439009.
