# Supplementary material for: Case Report: Whole exome sequencing identifies a novel frameshift insertion c.1325dupT (p.F442fsX2) in the tyrosine kinase domain of BTK gene in a young Indian individual with X-linked agammaglobulinemia
Source: F1000Res. 2017 Aug 31;5:2667. Originally published 2016 Nov 14. [Version 2] doi: 10.12688/f1000research.9472.2 (PMC5590079; doi:10.12688/f1000research.9472.2)
Supplement: Peer review history

# Peer review: Referee response for version 2

**DOI:** 10.5256/f1000research.13113.r25554

**Published:** 2017-09-07

**Authors:** Saharuddin Bin Mohamad

**Affiliations:** 1 Institute of Biological Sciences, Faculty of Science, University of Malaya, Kuala Lumpur, Malaysia

I am satisfied with the corrections and comments made by the authors.

I have read this submission. I believe that I have an appropriate level of expertise to confirm that it is of an acceptable scientific standard.

# Peer review: Referee response for version 1

**DOI:** 10.5256/f1000research.10201.r20086

**Published:** 2017-02-10

**Authors:** Saharuddin Bin Mohamad

**Affiliations:** 1 Institute of Biological Sciences, Faculty of Science, University of Malaya, Kuala Lumpur, Malaysia

XLA is a rare disease which gave this case report high value for being indexed. However I have some concerns regarding the report as below:

1. Since the patient was suspected with XLA and BTK gene is well known as the cause of the condition. Why Whole Exome Sequencing (WES) was conducted for this case? Why not conduct targeted sequencing for BTK since that would be much cheaper and easier to analyse. Since WES was conducted, I think authors should provide other results from WES analysis such as variant analysis, etc.
2. The results in Figure 1D for heterogeneous needs further explanation. It seems like the heterogeneous is not having two peaks at the asterisks position, but having two peaks at 5 positions downstream to the asterisks.
3. I would like to suggest evidence to be provided for the truncated BTK gene due to the mutation (Reverse transcriptase-PCR).
4. I would like to suggest the authors to referred to other database with bigger number of samples such as Exome Aggregation Consortium and 1000 Genomes database (not only BTKbase).

I have read this submission. I believe that I have an appropriate level of expertise to confirm that it is of an acceptable scientific standard, however I have significant reservations, as outlined above.

# Response

**Published:** 2017-03-02

**Authors:** Amit Rawat

**Affiliations:** Postgraduate Institute of Medical Education and Research, Chandigarh, India

Why Whole Exome Sequencing (WES) was conducted for this case?

 The differential diagnosis in a case of hypogammaglobulinemia with absent B cells would include X-linked agammaglobulinemia and autosomal recessive forms of hypogammaglobulinemia. X-linked agammaglobulinemia is caused by mutations in the BTK gene, but there is wide list of genes implicated in autosomal recessive forms of hypogammaglobulinemia. These include

*PIK3R1, GRB1, AGM7, IMD36, LRRC8A, KIAA1437, AGM5, BLNK, SLP65, AGM4*

*IGHM, MU, AGM1, CD79B, IGB, B29, AGM6, CD79A, IGLL1, IGO, IGL5, VPREB2, AGM2*

*AGMX2, XLA2, IMD6, IMD1, AGMX*1

 Therefore, a whole exome sequencing was used as diagnostic strategy in this case

 The variant found in the index case was also checked in the ExAC and not found to be reported there.

# Response

**Published:** 2017-07-26

**Authors:** Vinod Scaria

**Affiliations:** Institute of Genomics and Integrative Biology, Delhi, India

XLA is a rare disease which gave this case report high value for being indexed. However I have some concerns regarding the report as below:

**1. Since the patient was suspected with XLA and BTK gene is well known as the cause of the condition. Why Whole Exome Sequencing (WES) was conducted for this case? Why not conduct targeted sequencing for BTK since that would be much cheaper and easier to analyse. Since WES was conducted, I think authors should provide other results from WES analysis such as variant analysis, etc.**

**Clarification 1:** Targeted sequencing of BTK is not readily available in the India. In many cases, due to the unavailability of ready services, whole exome sequencing is an attractive alternative due to the speed, cost-effectiveness as well as the coverage of genes. Indeed the targeted sequencing for BTK was performed previously by another organization outside the country, which was reported negative, which prompted us to explore additional genes involved. In addition, number of genes have been previously implicated in agammaglobulinemia including PIK3R1, GRB1, AGM7, IMD36, LRRC8A, KIAA1437, AGM5, BLNK, SLP65, AGM4 IGHM, MU, AGM1, CD79B, IGB, B29, AGM6, CD79A, IGLL1, IGO, IGL5, VPREB2, AGM2, AGMX2, XLA2, IMD6, IMD1, AGMX apart from BTK.

The whole exome sequencing generated a total of 67437 variants in the patient, and 2046 and 891 variants for the mother and father respectively. Analysis of the variants are summarized in Table 1.

Analysis revealed four variants in genes related to agammaglobulinemia. Out of which three were with an allele frequency quite common in the Indian population. The BTK variant was previously not described in the variant frequency databases. The variants are summarized in Table 2.

**2. The results in Figure 1D for heterogeneous needs further explanation. It seems like the heterogeneous is not having two peaks at the asterisks position, but having two peaks at 5 positions downstream to the asterisks.**

**Clarification 2:** The asterisk position represents the site where ‘A’ base is inserted. The frameshift starts to be noticed after 1 peak due to AAA repeats. Please see Figure.

**3. I would like to suggest evidence to be provided for the truncated BTK gene due to the mutation (Reverse transcriptase-PCR).**

**Clarification 3:** We could not avail the RNA sample for the case for further studies. We have added a sentence in the discussion to highlight this caveat. Ample evidence suggests that the nonsense mutation at the codon Y425X, E441X, Q459X and Q497X is known to cause loss of kinase activity of BTK, which has been previously demonstrated using *in vitro* kinase activity assay in Japanese individuals (Hashimoto et al. 1996) . Since c.1325dupT (p.F442fsX2) lies in the vicinity of the above mentioned well studied codon positions, the effect of the mutation may be damaging to BTK.

Ref: Hashimoto S, Tsukada S, Matsushita M, et al.: Identification of Bruton’s tyrosine kinase (Btk) gene mutations and characterization of the derived proteins in 35 X-linked agammaglobulinemia families: a nationwide study of Btk deficiency in Japan. *Blood.* 1996;88(2):561–73. 8695804

**4. I would like to suggest the authors to referred to other database with bigger number of samples such as Exome Aggregation Consortium and 1000 Genomes database (not only BTKbase).**

**Clarification 4:** The suggestions have been incorporated in the revised manuscript. Databases such as ExAC, 1000genome and al-mena (Koshy et al. 2017) are mentioned in the manuscript.

Ref: Koshy R, Ranawat A, Scaria V. al mena: a comprehensive resource of human genetic variants integrating genomes and exomes from Arab, Middle Eastern and North African populations. Journal of Human Genetics. 2017 Jun 22.

# Response

**Published:** 2017-09-04

**Authors:** Vinod Scaria

**Affiliations:** Institute of Genomics and Integrative Biology, Delhi, India

There is bit correction in the total number of variations mentioned in comment box (See **Clarification 1)**. The whole exome sequencing generated a total of 67437 variants in the patient, and 61429 and 31074 variants for the mother and father respectively.

# Peer review: Referee response for version 1

**DOI:** 10.5256/f1000research.10201.r17566

**Published:** 2017-01-20

**Authors:** Mohamed Badawy Hassan Tawfik Abdel-Naser

**Affiliations:** 1 Faculty of Medicine, Ain Shams University, Cairo, Egypt

- The case report is interesting. 
- The authors unnecessarily repeated several sentences and phrases e.g. in the Introduction section the sentence "X-linked agammaglobulinemia (XLA) is a primary immunodeficiency disorder ...." has been repeated in the discussion section. Also, in the case report section the phrase "of the right knee joint". 
- The findings of the whole exome sequencing analysis should be regarded as interesting findings because a causal relationship with X-linked agammaglobulinemia needs additional cases and animal studies.

I have read this submission. I believe that I have an appropriate level of expertise to confirm that it is of an acceptable scientific standard.
